# Accuracy of non-invasive core temperature monitoring in infant and toddler patients: a prospective observational study

**DOI:** 10.1007/s00540-024-03404-7

**Published:** 2024-09-11

**Authors:** Tasuku Fujii, Masashi Takakura, Tomoya Taniguchi, Kimitoshi Nishiwaki

**Affiliations:** 1 Department of Anesthesiology, Nagoya University Hospital, 65 Tsurumai-cho, Showa-ku, Nagoya, 466-8550 Japan; 2 Department of Anesthesiology, Nagoya University Graduate School of Medicine, Nagoya, Japan

**Keywords:** Body core temperature, Infant, Non-invasive monitoring, Pediatrics

## Abstract

**Purpose:**

Careful perioperative temperature management is important because it influences clinical outcomes. In pediatric patients, the esophageal temperature is the most accurate indicator of core temperature. However, it requires probe insertion into the body cavity, which is mildly invasive. Therefore, a non-invasive easily and continuously temperature monitor system is ideal. This study aimed to assess the accuracy of Temple Touch Pro™ (TTP), a non-invasive temperature monitoring using the heat flux technique, compared with esophageal (Tesoph) and rectal (Trect) temperature measurements in pediatric patients, especially in infants and toddlers.

**Methods:**

This single-center prospective observational study included 40 pediatric patients (< 3 years old) who underwent elective non-cardiac surgery. The accuracy of TTP was analyzed using Bland–Altman analysis and compared with Tesoph or Trect temperature measurements. The error was within ± 0.5 °C and was considered clinically acceptable.

**Results:**

The bias ± precision between TTP and Tesoph was 0.09 ± 0.28 °C, and 95% limits of agreement were  – 0.48 to 0.65 °C (error within ± 0.5 °C: 94.0%). The bias ± precision between TTP and Trect was 0.41 ± 0.38 °C and 95% limits of agreement were  – 0.35 to 1.17 °C (error within ± 0.5 °C: 68.5%). In infants, bias ± precision with 95% limits of agreement were 0.10 ± 0.30 °C with  – 0.50 to 0.69 °C (TTP vs. Tesoph) and 0.35 ± 0.29 °C with  – 0.23 to 0.92 °C (TTP vs. Trect).

**Conclusion:**

Core temperature measurements using TTP in infants and toddlers were more accurate with Tesoph than with Trect. In the future, non-invasive TTP temperature monitoring will help perioperative temperature management in pediatric patients.

**Supplementary Information:**

The online version contains supplementary material available at 10.1007/s00540-024-03404-7.

## Introduction

Perioperative body temperature management is an important factor that affects clinical outcomes [1, 2]. The core body temperature in pediatric patients drops or rises more markedly than in adults because of the higher surface-area-to-weight ratio [3]. Despite careful body temperature management, intraoperative hypothermia or hyperthermia is common in pediatric patients. Intraoperative hypothermia is associated with major complications, such as risk of delayed recovery from anesthesia, shivering, myocardial ischemia, surgical site infections, coagulopathy, and increased blood loss [4–7]. In contrast, hyperthermia is associated with complications such as surgical site infection and tachycardia [8]. In pediatric patients, especially neonates and infants, hypothermia can induce serious pathophysiological disorders. Infants respond to hypothermia with a non-shivering metabolic response that increases oxygen consumption, glucose utilization, and metabolic rate. These lead to tissue hypoxia, hypoglycemia, and metabolic acidosis, resulting in physiological changes such as low arterial blood pressure, decreased cardiac output and cerebral blood flow, and pulmonary vasoconstriction [9]. Therefore, optimal intraoperative temperature management can improve postoperative outcomes, especially during pediatric anesthesia.

The gold standard for core temperature monitoring is the blood temperature measured using a pulmonary artery catheter. However, its general clinical use is limited, especially in pediatric patients, because of its size, invasiveness, and complications [10]. In a common intraoperative temperature measurement method, a temperature probe is inserted into the body cavity, such as the bladder, nasopharynx, esophagus, or rectum [11]. The bladder is not feasible, especially in neonates and infants, owing to the catheter size. Therefore, in pediatric patients, the intraoperative temperature is typically monitored using nasopharyngeal, esophageal, or rectal probes. Furthermore, Robinson et al. showed that esophageal temperature was the most accurate indicator of core temperature [12]. However, esophageal probe insertion requires general anesthesia and can cause clinically significant complications such as perforation, misplacement, and bleeding [13, 14]. Rectal probes can contribute to similar complications. Therefore, a non-invasive method to monitor temperature easily and continuously that can be used throughout the perioperative period, including in the awake state, would be ideal.

Recently, skin temperature measurement techniques over the temporal artery have been developed as alternatives to measuring core body temperature with non-invasive monitoring using the heat flux technique [15, 16]. Several studies have investigated one of the heat flux technologies, Temple Touch Pro™ (TTP; Medisim Co. Ltd., Israel), during general anesthesia in adults [17, 18]. However, the accuracy of core temperature measurement using TTP in pediatric patients has rarely been studied [19, 20], and no evidence exists in infants. Infants’ core and skin temperature differ from that of adults. Indeed, core temperature in infants is higher than that in adults, and the forehead skin temperature increases with age in toddlers [21]. Pediatric patients have a higher body surface area to mass ratio resulting in heat dissipation. We hypothesized that TTP accurately reflects the esophageal temperature (Tesoph) in infants and toddlers as it does in adults. We further hypothesized that TTP, a non-invasive temperature monitoring, would be more accurate than rectal temperature (Trect) measurements, which can be used in awake infants. Therefore, this study aimed to assess the accuracy and precision of TTP, a non-invasive temperature monitoring method, and compare it with esophageal and rectal temperature measurements in infants and toddlers.

## Methods

### Study design and patients

This single-center prospective observational study complied with the Declaration of Helsinki and was approved by the Nagoya University Hospital Ethics Committee (approval no.: 2022-0067) on June 1, 2022. This study was registered with the Japan Registry of Clinical Trials (jRCT1042220006) on April 16, 2022. Written informed consent was obtained from the parents of the pediatric patients. The study included 40 infants and toddlers aged < 3 years old who underwent elective non-cardiac surgery under general anesthesia between June 2022 and March 2024. Patients who used suppositories before measuring temperature were excluded.

### Measurements and outcomes

**Fig. 1:**
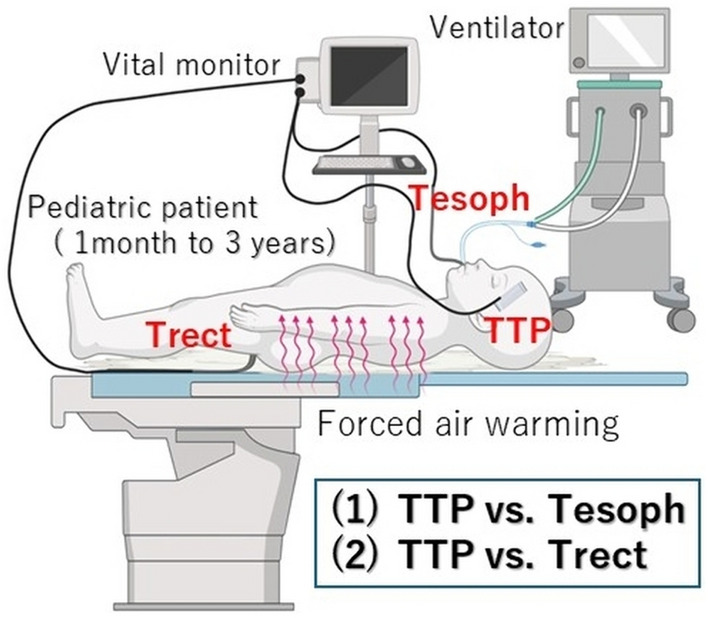
Overview of the study. This study assesses the accuracy between TTP and Tesoph or Trect in pediatric patients under general anesthesia. Illustrations are created using Biorender.com. *TTP* Temple Touch Pro, *Tesoph* esophageal temperature, *Trect* rectal temperature

Body core temperature, TTP, and reference sites (esophagus and rectum) were monitored immediately after the induction of general anesthesia (Fig. 1). A disposable temperature probe (ER400-9; Smiths Medical Japan, Tokyo, Japan) was placed in the distal esophagus for Tesoph, or approximately 2–3 cm via the anus for Trect without radiographic confirmation. A disposable sensor unit for the TTP (Medisim Co. Ltd., Israel) was attached to the temple over the temporal artery. Paired temperature values were recorded at 2-min intervals until the surgery was completed. After surgery, the core temperature data (TTP, Tesoph, and Trect) were collected on a bedside patient monitor (IntelliVue MX700; Philips, Amsterdam, Netherlands) for subsequent analysis. The agreement between TTP and esophageal or rectal temperatures was evaluated in infants and toddlers under general anesthesia. In this study, infants referred to babies aged 1–12 months, and toddlers to those aged 1–3 years.

### Statistical analysis

The sample size was estimated as follows owing to a lack of reference data for infants and toddlers. Analyses were performed using PASS 2024, version 24.0.2 (NCSS, LLC. Kaysville, Utah, USA) with a mean bias of 0.1, standard deviation (SD) of 0.25, and maximum allowable difference of 0.5 between two temperature measurements [22]. The required sample size was set to 414 paired measurements with 80% power and a significance level of 0.05. Assuming that 20–25 paired points of temperature measurement could be present in one patient, a total of 40 patients, including infants and toddlers, were required to account for a few dropouts.

As the primary outcome, the agreement and correlation between each core body temperature monitoring were assessed using the Bland–Altman analysis and the Spearman’s rank correlation coefficient: TTP vs. Tesoph and TTP vs. Trect. As a secondary outcome, the agreement and correlation between each core temperature monitoring method were evaluated in the infant patients. Similarly, the accuracy of core temperature measurements during laparoscopic surgery was evaluated. The Bland–Altman analysis provides the bias, SD (precision), and 95% limits of agreement (mean bias ± 1.96 SD) [23, 24]. Many studies on non-invasive body temperature monitoring [17–20], considered measurement errors within ± 0.5 °C between each temperature measurement clinically acceptable because ± 0.5 °C approximates normal circadian variation, and smaller deviations have never been shown to cause adverse events [25]. As the baseline characteristics of the pediatric patients, categorical variables are expressed as numeric values (proportion) and continuous variables as mean ± SD or median (interquartile range). Statistical significance was set at p < 0.05. All statistical analyses were performed using the R software, version 4.3.3 (The R Foundation for Statistical Computing, Vienna, Austria).

## Results

Forty patients were enrolled in this study, and all were analyzed, with no cases excluded. Patient characteristics are shown in Table 1. Supplementary Fig. 1 shows the raw data graphs of each body core temperature measurement.

**Table 1:** Patient characteristics

	Total (n = 40)	Infant (n = 20)	Toddler (n = 20)
Age (months)	14.1 ± 9.5	6.2 ± 3.6	22.0 ± 6.5
Height (cm)	72.7 ± 11.2	64.3 ± 8.4	81.1 ± 6.3
Weight (kg)	8.6 ± 2.9	6.7 ± 2.0	10.6 ± 2.2
Sex (male, %)	21 (52.5)	9 (45.0)	12 (60.0)
ASA-PS (1/2)	38/2	20/0	18/2
Surgical type			
SILPEC	17	11	6
Umbilical hernia	4	0	4
Polydactyly	4	0	4
Undescended testis	3	0	3
Soft tissue tumor	3	2	1
Others	9	7	2
Laparoscopic surgery (%)	21 (52.5)	15 (75.0)	6 (30.0)
Operation time (min)	71 ± 69	73 ± 59	69 ± 77
Anesthesia time (min)	160 ± 183	142 ± 67	179 ± 249

*ASA-PS* American Society of Anesthesiologists physical status, *SILPEC* single-incision laparoscopic-assisted percutaneous extraperitoneal closure

Values are presented as mean ± standard deviation or number (proportion, %) of patients

### Comparison of TTP and esophageal temperature

**Fig. 2:**
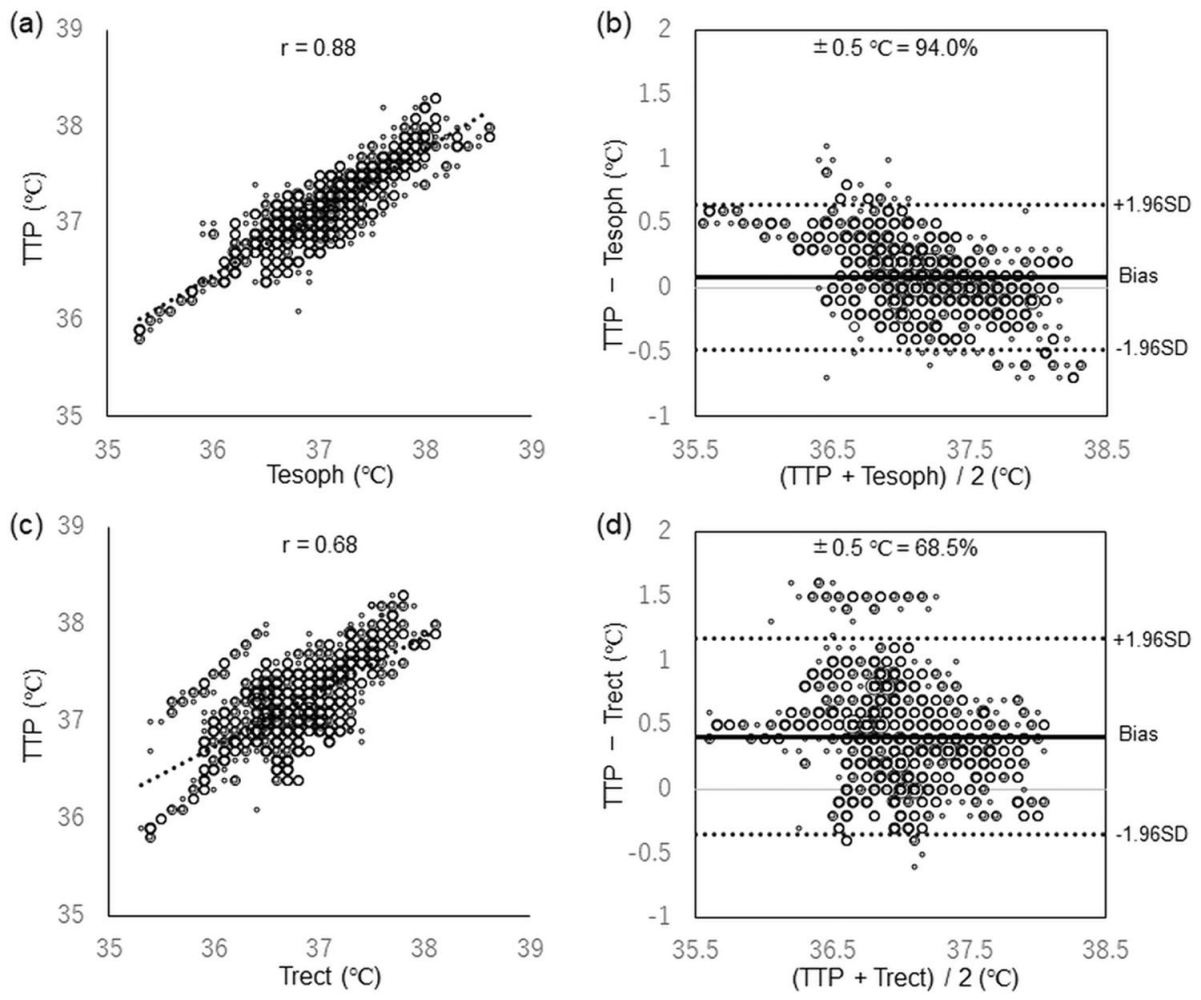
Comparison between TTP and Tesoph and TTP and Trect in infant and toddler patients. **a**, **c** Correlation coefficients for TTP versus Tesph and TTP versus Trect, respectively. **c**, **d** Bland–Altman analyses for TTP versus Tesoph and TTP versus Trect, respectively. *SD* standard deviation, *TTP* Temple Touch Pro, *Tesoph* esophageal temperature, *Trect* rectal temperature

We compared 1128 points between TTP and Tesoph for core body temperature measurements. The correlation coefficient between TTP and Tesoph was 0.876 (Fig. 2a). Based on Bland–Altman analysis, the bias ± precision of each measurement was 0.09 ± 0.28 °C with 95% limits of agreement within  – 0.48 to 0.65 °C (Fig. 2b). The temperature difference of each measurement within ± 0.5 °C was 94.0%.

**Fig. 3:**
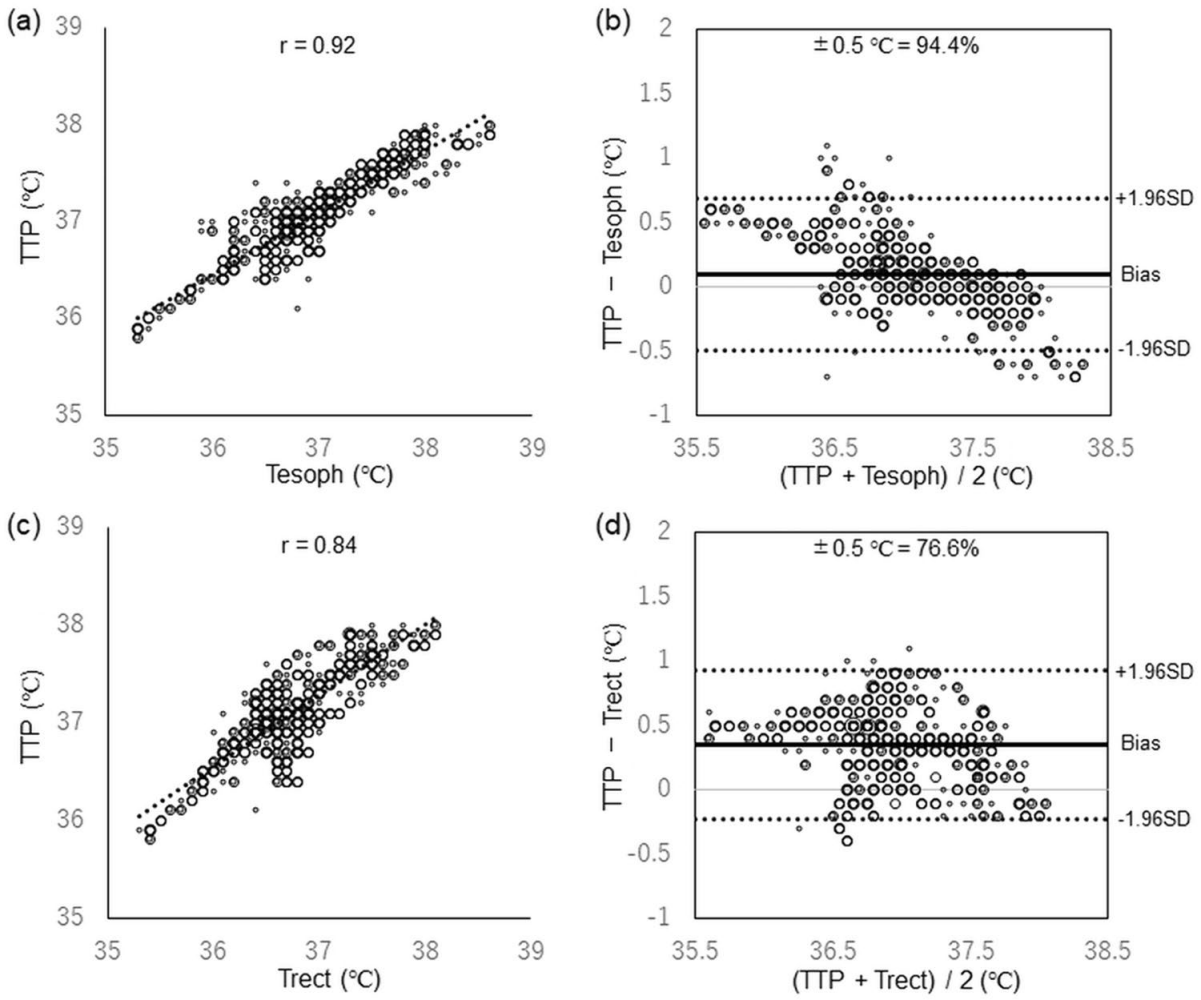
Comparison between TTP and Tesoph and TTP and Trect in infant patients. **a**, **c** Correlation coefficients for TTP versus Tesph and TTP versus Trect, respectively. **c**, **d** Bland–Altman analyses for TTP versus Tesph and TTP versus Trect, respectively. *SD* standard deviation, *TTP* Temple Touch Pro, *Tesoph* esophageal temperature, *Trect* rectal temperature

Analysis of 585 paired measurements in infants revealed a correlation coefficient of 0.919 (Fig. 3a) and bias ± precision of 0.10 ± 0.30 °C, with 95% limits of agreement within  – 0.50 to 0.69 °C between TTP and Tesoph (Fig. 3b).

### Comparison of TTP and rectal temperature

We compared 1,128 points between TTP and Trect for core body temperature measurements. The correlation coefficient between TTP and Trect was 0.685 (Fig. 2c). Based on Bland–Altman analysis, the bias ± precision of each measurement was 0.41 ± 0.38 °C with 95% limits of agreement within -0.35 to 1.17 °C (Fig. 2d). The temperature difference of each measurement within ± 0.5 °C was 68.5%.

In infants (n = 20), analysis of 585 points indicated a correlation coefficient of 0.838 (Fig. 3c) and bias ± precision of 0.35 ± 0.29 °C with 95% limits of agreement within  – 0.23 to 0.92 °C between TTP and Trect (Fig. 3b).

### Comparison between TTP and esophageal or rectal temperature under laparoscopic surgery

We compared 457 points in 21 cases between TTP and Tesoph or Trect for core body temperature measurements using Bland–Altman analysis. The bias ± precision with 95% limits of agreement were 0.04 ± 0.31 °C with  – 0.56 to 0.64 °C (TTP vs. Tesoph) and 0.48 ± 0.35 with  – 0.20 to 1.16 °C (TTP vs. Trect). The temperature difference within ± 0.5 °C were 89.9% (TTP vs. Tesoph) and 51.2% (TTP vs. Trect).

### Adverse events

In this study, complications related to each temperature measurement and adverse events owing to intraoperative hypothermia or hyperthermia were not observed.

## Discussion

This prospective study assessed the agreement between TTP and esophageal or rectal temperature measurements in infants and toddlers. We confirmed the clinical accuracy of TTP as an alternative to esophageal temperature measurements in pediatric patients within the clinically acceptable temperature range. We further demonstrated that TTP was more accurate with esophageal temperature than with rectal temperature. In the future, the results of this non-invasive temperature measurement will contribute to perioperative temperature measurement and management in pediatric patients, especially infants.

Esophageal temperature reflects core body temperature in pediatric patients most accurately and is widely accepted in perioperative clinical practice [12]. However, an ideal temperature measurement system should be able to measure core body temperature, even in awake patients. TTP is non-invasive because it can measure core temperature from the body surface and allows continuous monitoring of temperature in patients of any age or level of consciousness. No studies have evaluated non-invasive core temperature measurements in infants. Therefore, TTP can be considered a clinically useful perioperative core body temperature monitoring device because of its high accuracy in measuring esophageal temperature, not only in adults but also in infants and toddlers.

Nemeth et al. demonstrated an acceptably accurate TTP in pediatric patients compared with Tesoph [20]. They revealed a mean difference of  – 0.07 °C with 95% limits of agreement of  – 1.00 °C to 0.85 °C in pediatric patients with a mean age ± SD of 34.8 ± 25.2 months (range, 5 days to 6.9 years), a wider age range than our study population [20]. Sang et al. also investigated the accuracy of non-invasive zero-heat-flux thermometer similar to the TTP sensor, SpotOn™ sensor (3 M™ Bair Hugger™ sensor, 36,000, 3 M Medical, USA), compared with Tesoph in non-infant pediatric patients with mean age ± SD of 45 ± 16 months (range, 1–8 years) [26]. They showed a bias of -0.07 °C and 95% limits of agreement of  – 0.41 to 0.28 °C [26]. Our study also showed that the bias of each measurement was 0.09 °C with 95% limits of agreement within  – 0.53 to 0.70 °C and the proportion of error within ± 0.5 °C was 92.2% in younger pediatric patients (mean age ± SD of 14.0 ± 9.5 months). Therefore, we confirmed the clinically acceptable accuracy of TTP compared with esophageal temperature in pediatric patients, especially in infants and toddlers.

Zeiner et al. assessed the agreement between non-invasive thermometry, using a Tcore™ (Dräger, Drägerwerk AG & Co. KG, Lübeck, Germany) and Trect in pediatric patients with a mean age of 26.7 months [27]. They indicated a bias of 0.41 °C and limits of agreement of  – 0.74 to 1.57 °C between the two measurements [27]. Our study showed that the bias of each measurement was 0.41 °C with 95% limits of agreement within  – 0.35 °C to 1.17 °C and the proportion of error within ± 0.5 °C was 65.7% between TTP and Trect. In this study, TTP was more accurate with esophageal temperature than rectal temperature. Rectal temperature is less reliable because it lags significantly behind the core temperature in response to rapid thermal changes owing to poor perfusion [25]. In addition, our results of the temperature accuracy under laparoscopic surgery in pediatric patients showed that the mean difference between TTP and Tesoph was 0.06 °C, which was within the clinically acceptable range. However, the bias between TTP and Trect was 0.50 °C, which is difficult to be accepted clinically. Therefore, it was suggested that TTP may have a broader clinical utility.

Nemeth et al. reported the occurrence of a minor skin lesion (superficial epidermal excoriation) owing to accidental removal of the sensor [20]. In our study, even in infants, only a few cases of temporary skin redness were noticed after the TTP sensor removal, similar to the ECG sensor; however, no skin lesions required intervention. The TTP sensor is considered safer than probes inserted into body cavities such as the esophagus or rectum. Furthermore, zero-heat-flux thermometer, SpotOn™, includes a heating component, which is initially preheated to equilibrium with the skin surface temperature. Therefore, manufacturers warn not to apply the sensor to fragile skin. TTP sensors, however, do not actively heat, but rather utilize local temperature and heat flow readings to estimate the core temperature. Therefore, we assume the TTP to be safer in infants, especially in awake conditions.

This study had several limitations. First, the study was conducted under general anesthesia. General anesthesia causes iatrogenic vasodilation, which restricts the thermal response of vasoconstriction in the periphery and leads to the redistribution of heat from the core to the periphery [28]. Additional studies are required to evaluate whether TTP is accurate in pediatric patients with several perioperative conditions, such as during awake state. However, our unpublished data compared TTP and axillary temperature in a small number of awake pediatric patients (n = 9) in the intensive care unit. The results showed that the bias and SD between both measurements was -0.07 °C in a mean age of 1.6 ± 1.8 months, and TTP may be clinically useful even under awake conditions in intensive care units. Second, the esophageal temperature probe was placed in the distal esophagus, and the specific insertion length was not defined. Zhong et al. showed that nasopharyngeal thermometers accurately measured core body temperature when the probe was inserted at the optimal location of an appropriate insertion length, depending on age [29]. However, in this study, the accuracy of the TTP and Tesoph measurements was clinically acceptable. Third, in this study, body temperature changes during surgery were small owing to strict temperature control with forced-air warming system. Therefore, we were unable to evaluate the tracking ability between each measurement method. In the future, it is necessary to evaluate the tracking ability in situations in which the body temperature changes significantly, such as before and after cardiopulmonary bypass. Finally, in this study, most pediatric patients had APA-PS1, which indicated good general conditions, including growth and development. Further research is required to determine whether core temperature measurements using the TTP sensor can be applied clinically in neonates and premature infants.

To summarize, this study demonstrated that the core body temperature evaluated using TTP in infants and toddlers had higher accuracy with esophageal than rectal measurements within the clinically accepted body temperature range. Further studies are warranted in extreme temperature ranges and changes to allow for more clinical applications of TTP in pediatric patients.

## Supplementary Information

Below is the link to the electronic supplementary material.

Supplementary file1 (JPG 497 KB) Supplementary Figure 1: The raw data graphs of each body core temperature measurement during general anesthesia. Each graph should show one line per patient (x-axis: time, y-axis: body temperature). Red line indicates infants and black line indicates toddlers. Tesoph, esophageal temperature; TTP, Temple Touch Pro; Trect, rectal temperature

## Data Availability

The datasets generated and/or analyzed during the current study are available from the corresponding author on reasonable request.
