# Supplementary figures and images for: Accuracy of non-invasive core temperature monitoring in infant and toddler patients: a prospective observational study
Source: J Anesth. 2024 Sep 11;38(6):848–54. doi: 10.1007/s00540-024-03404-7 (PMC11584424; doi:10.1007/s00540-024-03404-7)

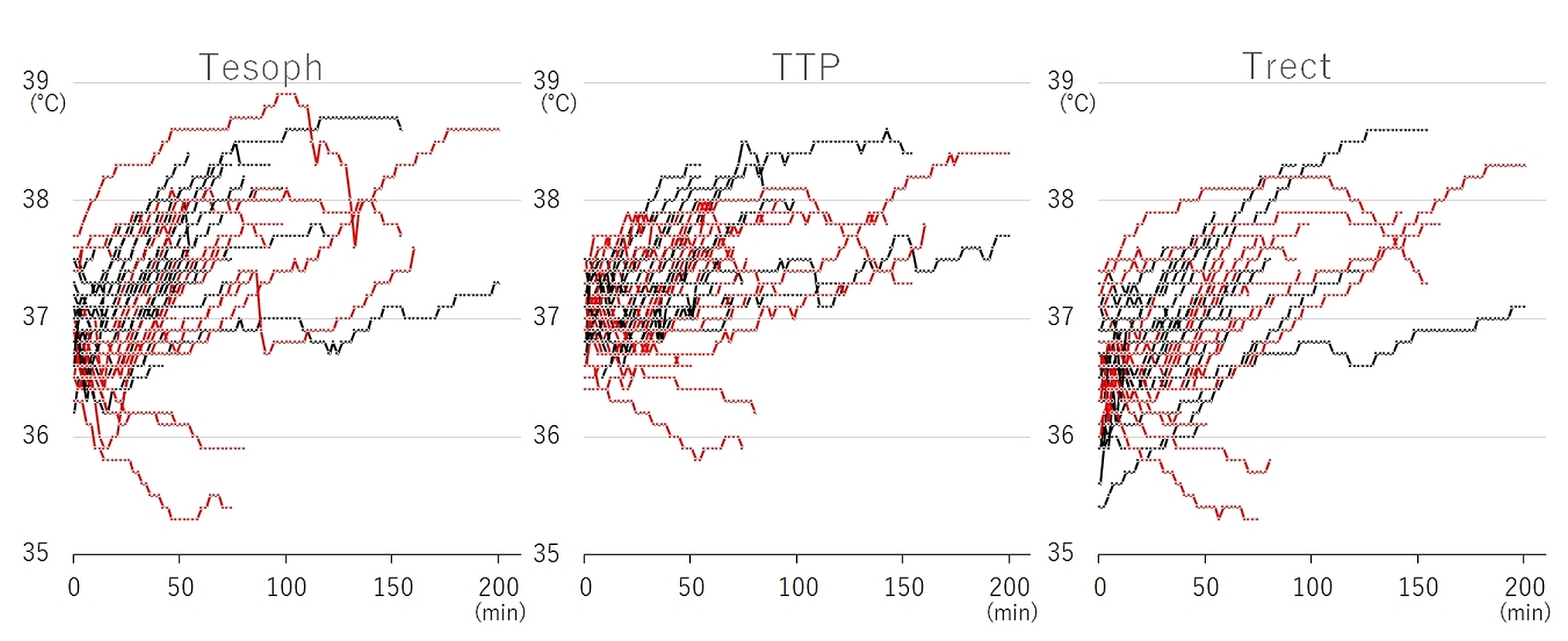

Supplement: Supplementary file 1 — Supplementary file1 (JPG 497 KB) Supplementary Figure 1: The raw data graphs of each body core temperature measurement during general anesthesia. Each graph should show one line per patient (x-axis: time, y-axis: body temperature). Red line indicates infants and black line indicates toddlers. Tesoph, esophageal temperature; TTP, Temple Touch Pro; Trect, rectal temperature [file 540_2024_3404_MOESM1_ESM.jpg]
